# Supplementary material for: Elevation and Human Disturbance Interactively Influence the Patterns of Insect Diversity on the Southeastern Periphery of the Tibetan Plateau
Source: Insects. 2024 Sep 4;15(9):669. doi: 10.3390/insects15090669 (PMC11432195; doi:10.3390/insects15090669)
Supplement: Supplementary file 1 [file insects-15-00669-s001.zip › insects-3146943-supplementary.pdf]

Supplementary Materials

Table S1. Insect community composition by family on the southeastern margin of the Tibetan Plateau.

Order	Family	Individuals
Orthoptera	Acrididae	299
Orthoptera	Tettigoniidae	103
Orthoptera	Tetrigidae	2
Orthoptera	Gryllidae	21
Orthoptera	Gryllotalpidae	2
Hemiptera	Pentatomidae	123
Hemiptera	Coreidae	421
Hemiptera	Alydidae	4
Hemiptera	Pyrrhocoridae	39
Hemiptera	Plataspidae	89
Hemiptera	Reduviidae	17
Hemiptera	Berytidae	27
Hemiptera	Scutelleridae	10
Hemiptera	Miridae	86
Hemiptera	Nabidae	1
Hemiptera	Tingidae	25
Hemiptera	Aleyrodidae	75
Hemiptera	Cicadidae	11
Hemiptera	Membracidae	20
Hemiptera	Cicadellidae	88
Hemiptera	Cercopidae	300
Hemiptera	Fulgoridae	20
Hemiptera	Aphidoidea	359
Hemiptera	Margarodidae	10
Hemiptera	Coccoidea	10
Coleoptera	Chrysomelidae	1229
Coleoptera	Curculionidae	79
Coleoptera	Attelabidae	8
Coleoptera	Coccinellidae	290
Coleoptera	Cerambycidae	12
Coleoptera	Carabidae	13
Coleoptera	Tenebrionidae	68
Coleoptera	Pselaphidae	1
Coleoptera	Buprestidae	5
Coleoptera	Canthqqrudae	1
Coleoptera	Melolonthidae	88
Coleoptera	Rutelidae	27
Coleoptera	Cetoniidae	4
Coleoptera	Geotrupidae	2

Coleoptera	Meloidae	37
Coleoptera	Elateridae	137
Coleoptera	Lucanidae	2
Coleoptera	Hispidae	4
Coleoptera	Cicindelidae	1
Coleoptera	Staphylinidae	2
Coleoptera	Dytiscinae	4
Coleoptera	Scolytudae	1
Diptera	Muscidae	43
Diptera	Syrphidae	57
Diptera	Tephritidae	21
Diptera	Culicidae	16
Diptera	Calliphoridae	65
Diptera	Tipulidae	21
Diptera	Bibionidae	3
Diptera	Tabanidae	8
Diptera	Alisidae	14
Diptera	Stratiomyidae	2
Hymenoptera	Formicidae	472
Hymenoptera	Vespidae	16
Hymenoptera	Sphecidae	4
Hymenoptera	Ichneumonidae	36
Hymenoptera	Arthropoda	2
Hymenoptera	Polistidae	1
Neuroptera	Chrysopidae	27
Neuroptera	Myrmeleontidae	16
Neuroptera	Mantispidae	1
Neuroptera	Hemerobiidae	4
Lepidoptera	Pieridae	15
Lepidoptera	Lycaenidae	5
Lepidoptera	Hesperiidae	9
Lepidoptera	*Nymphalidae*	4
Lepidoptera	Satyridae	12
Lepidoptera	Papilionidae	6
Trichoptera	Phryganeidae	50
Phasmatodae	Phasmatidae	5
Dermaptera	Labiduridae	10
Mantodea	Mantidea	10
Odonata	Libellulidae	4
Odonata	Aeshnidae	1
Blattaria	Blattidae	4
